# Real estate valuation with multi-source image fusion and enhanced machine learning pipeline

**DOI:** 10.1371/journal.pone.0321951

**Published:** 2025-05-19

**Authors:** Lin Deng

**Affiliations:** Department of Civil and Environmental Engineering, Hong Kong University of Science and Technology, Hong Kong, China; Zhengzhou University, CHINA

## Abstract

The automated valuation model (AVM) has been widely used by real estate stakeholders to provide accurate property value estimations automatically. Traditional valuation models are subjective and inaccurate, and previous studies have shown that machine learning (ML) approaches perform better in real estate valuation. These valuation models are based on structured tabular data, and few consider integrating multi-source unstructured data such as images. Most previous studies use fixed feature space for model training without considering the model performance variation brought by various feature configuration parameters. To fill these gaps, this study uses Hong Kong as a case study and proposes an enhanced ML-based real estate valuation framework with feature configuration and multi-source image data fusion, including exterior housing photos, street view and remote sensing images. ^‌‌^Eight ML regressors, namely, Random Forest, Extra Tree, XGBoost, Light Gradient Boosting Machine (LightGBM), K-Nearest Neighbors (KNN), Support Vector Regression (SVR), Multilayer Perceptron (MLP), and Multiple Linear Regression (MLR) are used to formulate ML pipelines for training. The SHapley Additive exPlanations (SHAP) method is used to examine the effects of images on housing prices. The experimental results show that the model performances using different feature configuration parameters are significantly different, indicating the necessity of feature configuration to obtain more accurate and reliable predictions. Extra Tree performs significantly better than other models. Half of the top 10 significant features are image features, and incorporating multi-source image features can improve property valuation accuracy. Nonlinear associations exist between image features and housing prices, and the spatial distribution patterns of image feature values and corresponding SHAP main effects vary significantly from the city centre to the suburbs. These findings contribute to a better understanding of AVM development with image fusion and the nonlinear associations between image features and housing prices for public authorities, urban planners, and real estate developers.

## Introduction

The automated valuation model (AVM) is a mathematically based computer program designed to estimate the prices of properties [1]. To develop an AVM, the developers must first identify the influencing factors of housing prices and then build a statistical model to mathematically describe the relationships between the factors and housing prices based on big housing transaction data. Compared with traditional human-based valuations, AVM is more accurate and much cheaper and, therefore, has been commonly used in the real estate industry for property valuation, supplementing or replacing the work of human valuers.

Two groups of factors mainly influence housing prices: macro-level and micro-level [2]. Macro-level factors are mostly time-dependent housing market factors, including inflation, interest, unemployment, and gross domestic product (GDP) [3]. Micro-level factors include property characteristics and locational features [4]. The property characteristics refer to view orientation, number of rooms, floor level and area, building age, etc. Locational features are mainly extracted based on the geographic location of the house, such as geographic coordinates, distance to the city centre or central business districts (CBDs), density, diversity, quality and accessibility of neighbourhood amenities, sociodemographic status, crime rate, and built and natural environment.

Traditional property valuation models include cost, income, and sale comparison approaches [1]. The cost approach is an indirect method, calculating the value of a property as replacement cost less depreciation plus the market-derived land value. The income approach estimates the market value of a property as the net operating income divided by the capitalization rate. The sale comparison approach includes: (1) **comparable sale model** that estimates property values based on small samples of properties with similar characteristics. (2) **direct market model** that describes property values as a function of a property’s location and physical attributes. The hedonic pricing model (HPM) is often used to depict the relationship between the housing price and its determinants [5] using elasticity with semi-log or log-log transformations. Traditional models are well-explanatory and interpretable but have limited capability [6]. The HPM excels in explaining the elasticity of real estate economics, but it has low property valuation accuracy and cannot capture the nonlinear associations [7].

With the exponentially increasing housing transaction volume, machine learning (ML) and deep learning (DL) models have been applied to property valuation [8] in a neighbourhood, city, or country. ML and DL models outperform traditional models in extracting information from big data, measuring the nonlinear relationships between property value and its characteristics, and feature selection [9, 10]. Two primary strands with ML and DL applications emerge: **(1) Method-oriented studies** that adopt novel methods to showcase improved prediction accuracy [6,11–14], and **(2) Features-oriented studies** that explore the significance of specific features in enhancing price predictions and delve into the economic rationale behind their relevance [15–21]. The most common ML models used in previous studies include tree-based models, k-nearest neighbour (KNN), support vector machine (SVM), multilayer perceptron (MLP), deep artificial neural networks (DANN), etc. A detailed overview using ML and DL models for housing price prediction is provided in the review papers [22–24]. Among these ML and DL models, tree-based models usually perform the best due to ensemble learning strategies.

Among previous property valuation studies, most features are structured data and easy to quantify, except for the built and natural environment that affects homebuyers’ willingness to pay for the properties and are complex to convert into numerical values. Given recent advancements in deep learning (DL) based computer vision techniques, researchers can now extract built and natural environment features from multiple types of images [25,26]. These studies show that image-based features contribute to the accuracy of housing price predictions. Integrating various types of images into AVM development to achieve higher valuation accuracy is deemed imperative. Despite the significant insights of the existing studies, some research gaps remain. First, studies on AVM development with multi-source image fusion and comparing the effects of different image features on housing prices are still limited. Second, most studies use single feature configuration space for model training, and little is known about how one should make decisions regarding generating the best-performing features and how the resultant image-based features may affect the model performance based on the choices made. The effects of feature configuration parameters on model performances should be thoroughly investigated.

In this study, an enhanced ML-based residential property valuation framework is proposed by (1) fusing the multi-source images of exterior estate photos, street view images, and remote sensing images, (2) identifying the feature configuration parameters to formulate a series of ML pipelines, (3) using a server-client based distributed computing strategy to accelerate the ML pipeline training process, (3) evaluating the ML pipelines’ performances to analyze the effects of feature configuration and selection, and ML models, (4) enhancing the interpretability of the ML-based approach by analyzing the model-based global feature importance and the SHAP-based local feature importance.

## Literature review

This section critically reviews studies on real estate valuation with multi-source image fusion, focusing specifically on multi-source image data and features, and image feature extraction. Table 1 summarizes the studies regarding research scope, image data, image sampling configuration, image processing, configuration, prediction model, and research results.

**Table 1:** Summary of studies on image-based property valuation.

References	Research scope	Image data	Image sampling configuration	Image processing configuration	Prediction model	Research results
[27]	England and Wales: 49,603 properties since 2011	70,059 GSV images	**Sampling interval:** 100 m	**SP:** BPNN	MLR	SP variables impact house prices more than some objective urban environment attributes.
[28]	Tokyo, Japan: 17,552 properties for sale and 137,851 properties for rent from 2006 to 2015	Two satellite images on April 30, 2008, and October 13, 2013	**Spatial resolution:** 1.5 m	**RSI:** NDVI	MLR	A 10% increase in average scattered greenery within 100 m of a property increases the property price by 2 to 2.5%
[29]	Shanghai, China: 53,445 records in 2019	25,726 BSV images	**Sampling interval:** 50 m **Sampling range:** 1000 m **Horizon view:** parallel to the street centerline **Vertical view**: 0° **FOV:** 120° **Image size:** 640×360 **Time:** summer and fall	**SP:** RF, DT, VS, GB, AdaBoost **VI:** Pretrained Pyramid Scene Parsing Network (PSPNet) based on ADE20K dataset	MLR, SAC	Perception variables and view index variables increase the R squared by: 0.005 and 0.003 from the baseline (0.791) with OLS model
[30]	Amsterdam: 12,352 records on July 9, 2020; 11,798 records on November 3, 2020;	10,993 GSV images	**Sampling interval:** 100 m **Sampling range:** 100 m **Horizon view:** 0°, 90°, 180°, 270° **Image size:** 640×640	**VI:** Pretrained DeepLabV3 on the Cityscapes dataset	MLR, GWR	View index variables increase the R squared by 0.001–0.002 (MLR) and 0.005–0.01 (GWR)
[31]	Hachioji city, Japan: 800 records from 2016 to 2019	9600 GSV images of the selected properties (12 images per property)	**Horizon view:** every 30 degrees from 0° to 360° **Time:** August 2021	**VI:** Pretrained Hierarchical Multi-Scale Attention Deep Neural Network on the Mapillary and Cityscapes dataset	MLR	View index variables increase the R squared by 0.007 (OLS)
[32]	Shanghai, China: 53,445 records in 2019	25,726 BSV images	**Sampling interval:** 50m **Sampling range:** 1000m **Horizon view:** parallel to the street centerline **Vertical view:** 0° **FOV:** 120° **Image size:** 640×360	**SP:** RF **VI:** Pretrained PSPNet on the ADE20K dataset	MLR, SAC, GWR	SP and VI variables increase the R squared by 0.005 and 0.013 (OLS), 0.052 and 0 (SAC), 0.158 and 0.166 (GWR)
[33]	Netherlands: 1,844 records from April to June in 2020	Over 40,000 photos of the rental offers	\	**VT:** Pretrained CNN for classification	MLR, RF	Image and text variables increase the R2 by 0.02 (MLR) and 0.03 (RF)
[34]	London: 137,132 records from 2013 to 2015	1,154,849 geotagged Flicker images	**Sampling range:** 800 m	**VT:** Pre-trained Place365 CNN based on Flicker dataset	MLR, RF, GB	Image variables increase the R2 by 0.051 (MLR), 0.046 (RF) and 0.019 (GBM)
[35]	Zhengzhou, China: 499 records on 20 December, 2020	7,994 BSV images	**Sampling interval:** 50 m and 200 m **Sampling range:** 360/720/1080/2160 m **Horizon view:** 0°, 90°, 180°, 270° **Time:** 23 July, 2020	**VI:** Fully Convolutional Neural Network (FCN-8s)	\	The index of green space in old urban areas is higher than that in the new development zone of eastern Zhengzhou
[36]	Shenzhen, China: 2,296 residential communities	1,150,932 BSV images at 287,733 locations, 63,775 photos from online real estate websites	**Sampling interval:** 50 m **Sampling range:** 1000 m **Horizon view:** 0°, 90°, 180°, 270° **Vertical view:** 0°	**VI:** SegNet	MLR, RF, GWR	Visual contact with green space has positive impacts on housing prices
[37]	Beijing, China: 20,785 records in May 2021. Chongqing, China: 20,098 records in May 2021	64,569 BSV panorama images in Beijing, 38,987 BSV panorama images in Chongqing	**Sampling interval:** 50m **Sampling time:** September 2019	**VI:** DeepLabV3+	MLR, MGWR	The green view index is the most critical visual index affecting house prices and is positively correlated with house prices
[38]	Shanghai, China: 40,159 records in 2019	25,726 BSV images	**Sampling interval:** 50 m **Sampling range:** 1000 m **Horizon view:** parallel to street centerline **Vertical view:** 0° **FOV:** 120° **Image size:** 640×360	**SP:** SVM, RF, DT, GB. **VI:** PSPNet	MLR	SP variables and VI variables increase the R squared by 0.008 and 0.004 from the baseline model
[39]	Shenzhen, China: 12,137 records as of January 2021	BSV images; Luojia-1 Night-time light imagery	**Sampling interval:** 50 m **Sampling range:** 1000 m **Horizon view:** 0°, 90°, 180°, 270°	**VI:** SegNet, NDVI of 1000 m buffer	MLR, XGBoost	The green view index has significant and positive effects on housing prices
[40]	Taipei City and New Taipei City: 92,857 records from 2017 to 2018	92,857 Google satellite images	**Image size:** 640×640	**FV:** Spatial Transformer Network (STN)	MLR, XGBoost, LightGBM	Image features extracted from satellite maps with STN can improve model performance
[41]	New York: 1,404 records from 2010 to 2017	90,382 GSV images	**Sampling range:** 100 m **Horizon view:** north, south, east, and west **Time:** between April and September	**VI:** Python 3.7 OpenCV library	MLR	A positive and statistically significant impact exists on commercial building transaction prices and rents
[42]	Guri City, South Korea: 3,007 records	3,007 property photos	**Image size:** 150×150	**FV:** CNN	MLP	It is useful to use both photographs and metadata to enhance the accuracy of house price estimation
[10]	Greater Boston Area: 21,928 records	125,000 house photos and about 47,000 GSV images	**Sampling interval:** 100 m **Sampling range:** 50 m **Horizon view:** eight angles from 0° to 360°	**FV:** ResNet19	MLR, GBM	House photos and street view images increase R2 by 0.01 and 0.04, respectively
[43]	16 large and medium-sized cities in China: 79,212 records in November 2018	218,757 GaoDe multi-scale satellite images	\	**FV:** VGG16, Inception V3, Resnet18 for classification	MLR, SVM, MLP	Satellite images increase the R2 by 0.0240 (MLR), 0.0444 (SVM), and 0.0295 (MLP)
[44]	Los Angeles: 108,571 records; Boston: 94,892 records	110,998 GSV images collected from 56 cities among 28 countries	**Sampling interval:** 50 m **Horizon view:** four directions	**SP:** pretrained CNN	MLR, SAC, GWR	SP variables increase the R2 by 0.02 (MLR) in Los Angeles and 0.35 in Boston (MLR)
[45]	Philadelphia: 15,815 records	15,815 GSV images	**Horizon view:** only contains the front view of the house	**VT:** pretrained PSPNet, pretrained VGG16 of Place365 and deep expectation	BRT	Fusing metadata and expected levels of property values derived by image features has the highest R2
[46]	London: 6,110 records in 2011	112,650 front-facing street images from GSV API and CityEngine software	**Sampling interval:** median of the street edge between two junctions **Horizon view:** front view **Image size:** 256×256	**VT:** pretrained VGG-16, ResNet-50, Inception-V3, Xception on ImageNet data	MLR, spatial regression model	The quality of urban street frontage can be an important housing price determinant
[47]	New York and Massachusetts: 20,000 records in 2016	Indoor images, outdoor images (property photos and GSV images), Google satellite images	\	**FV:** pretrained ResNet152-hybrid1365 architecture on ImageNet and Places365 datasets	MLR, XGBoost, LightGBM, CATBoost	Image features contribute to the overall predictive power
[48]	Shanghai, China: 2547 properties in 2018	84,520 panoramic SVIs. GaoFen-1 data	**Sampling interval:** 50 m **Image size:** 1024×290 **Time:** August and September 2017 **Spatial resolution:** four multispectral bands at an 8 m spatial resolution and one panchromatic band at a 2 m spatial resolution.	**VI:** pretrained DeepLabv3 on the Cityscapes dataset; **RSI:** SVM for urban green (UG) and water (UW) coverage rate calculation within a 400 m radius buffer	MLR, XGBoost, RF, GBR	View index increase R2 from 0.6722 to 0.6971, and UG+UW increase R2 from 0.6722 to 0.6987
[49]	Middle ring road area in Shanghai, China: 1395 private neighborhoods in late 2016	Approximately 230,000 BSV images from 69,137 sample sites	**Sampling interval:** 40 m **Sampling range:** 1000 m **Horizon view:** 0°, 90°, 180°, 270° **Vertical view:** 0° **Image size:** 480×360	**VI:** SegNet	MLR	Human-scale daily accessed greenery is of great significance for housing price prediction
[50]	London, UK: 130,557 records	111,701 images for both the street image dataset and the aerial image dataset	**Sampling interval:** median of the street edge **Horizon view:** 120° **Image size:** 256 x 256	**FV**: CNN	MLR, GAM, XGB	Image features increase the R2 by 0.0512 (MLR), 0.0568 (GAM), and 0.0645 (XGB)
[51]	Ohio, US: 59,352 records in 2000	59,352 GSV images	**Sampling interval:** median of the street edge **Horizon view:** face the home, and surroundings at 90° interval **Image size:** 800 x 200	**VT:** SVM	MLR	The walkability features from images can better capture variations in home sale prices
[52]	Wuhan, China: 2349 records	11 Luojia1–01 satellite images	**Spatial resolution:** 130 m resolution	**RSI:** digital values	MLR, GWR	A strong linear positive correlation exists between Wuhan’s housing prices and night-time lights.
[53]	Beijing, China: 3,917 records; Shanghai, China: 2,915 records;	71,332 and 91,759 BSV panoramas in Beijing and Shanghai	**Sampling interval:** 100 m **Sampling range:** 800 m **Time:** between March and October 2015	**VI:** pretrained PSPNet based on MIT ADE20K dataset	MLR	The green view index and sky view index can significantly affect housing prices
[54]	Zillow: 9,000 records	More than 200k images of home interiors and exteriors	\	**VT:** DenseNet	SVR with radial basis function as the kernel	Visual information of the real estate photos reduces the median error rate by 2.4% compared to the benchmark with only metadata
[55]	Beijing, China: 22,331 records in 2014	208,746 Tencent SVI images	**Sampling interval:** 100 m **Sampling range:** 400 m **Horizon view:** six directions from 0° to 360° **Image size:** 600 x 600 **Time:** from April to October in 2014	**VI:** pretrained SegNet on the CamVid dataset (a driving photo dataset)	MLR	An improvement in GVI can significantly increase the nearby housing prices
[56]	Wuhan, China: 400 records from February 22 to October 31, 2010	Two ETM+ images on September 17 and October 13, 2010	**Spatial resolution:** 30m	**RSI:** mathematical equations	SLM	A 1% increase in the thermal environmental index and vegetation coverage index decreases the housing value by 54.54 and 33.75 RMB/m^2^
[57]	San Jose, US: 3064 records. Rochester, US: 1500 records	3064 property photos in San Jose and 1500 property photos in Rochester	\	**FV:** GoogleNet	Bidirectional-LSTM	Location is relatively more important than the visual features in the realtor business

Note: SP=Subjective perception. VI=View index. VT=View type. RSI=Remote sensing index. FV=Feature vectors. DT=Decision tree. BPNN=Backward Propagation Neural Network. VS=Voting selection. MLR=Multiple linear regression. SAC=Spatial autocorrelation. GWR=Geographically weighted regression. RF=Random Forest. GB=Gradient boosting. GBR=Gradient boosting regression. MGWR= Multiscale geographically weighted regression. MLP=Multiple layer perception. GBM=Gradient boosting machine. SVM=Support vector machine. GAM=Generalized additive model. SVR=Support vector regression. SLM=Spatial lag model. LSTM=Long short-term memory.

### Multi-source image data and features

**Fig 1:**
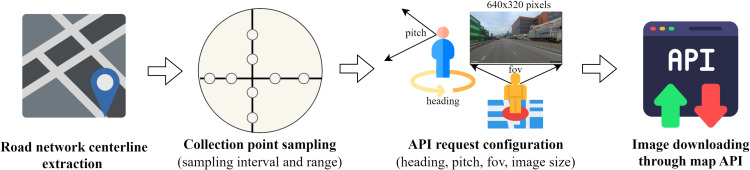
Procedures of SVI collection.

An image is worth a thousand words. Three types of images are most often used for hedonic analysis: interior and exterior housing photos, street view images, and remote sensing images. User-generated images are also explored for housing price estimation [34]. There are two types of street view images in a sampling point: (1) single-view images that have specific headings and (2) panoramic images that provide a 360-degree view of the surrounding environment [58]. Fig 1 shows street view image collection procedures, including road network centerline extraction, collection point sampling (sampling interval and range), API request configuration (heading, pitching, field of view, image size), and image downloading through map API. Remote sensing images include satellite images [28,43,56] and nighttime light (NTL) images [52]. They can reflect the landscape metrics and human activities near the property. Interior and exterior housing photos show the houses’ inner furnishing and outer appearance, which is significant in formulating the market value [54].

Image features can be subjective and objective. **Subjective features** refer to human perceptions of the surrounding environment [59], including greenness, walkability, safety, imageability, enclosure, and complexity [38], lively score [44], and safety score [60]. A crowdsourced dataset called Place Pulse is commonly used as the training dataset to derive the perception score [61]. Since the Place Pulse dataset does not include cities in mainland China, some researchers built the online survey and invited participants to select a preferred photo from two random street view images in response to questions such as “Which place looks greener?” [32]. The pairwise preference is then transformed into a perception score using the Microsoft TrueSkill rating algorithm [62]. The labelled images will be split into training and test sets to derive the pre-trained model to predict the perceptions of the remaining images [29]. **Objective features** include view index, view type, remote sensing index, and feature vector. View index is represented as the proportion of specific elements using semantic segmentation techniques, such as green view index, building view index, and sky view index [31,36]. View type refers to the classification results of images. View types of street view images include housing price levels [10,45], kind of view [46,33], and scene categories[34,51]. View types of remote sensing images can be high or low property price zones [43]. Housing photo-based view types include indoor attribute categories [47] and luxury level [54]. Remote sensing index includes normalized difference vegetation index (NDVI) [28,63], urban green and water coverage rate [48], thermal environmental index and vegetation coverage index [56], and NTL intensity [52,64]. Feature vectors are numerical vectors extracted by convolution neural networks (CNNs) and are usually combined with other numerical features to formulate the final feature space[50,42]. Despite the substantial contributions of these studies, they do not thoroughly examine a house’s built and natural environment from the perspective of exterior housing appearance, street and aerial view.

### Image feature extraction

**Fig 2:**
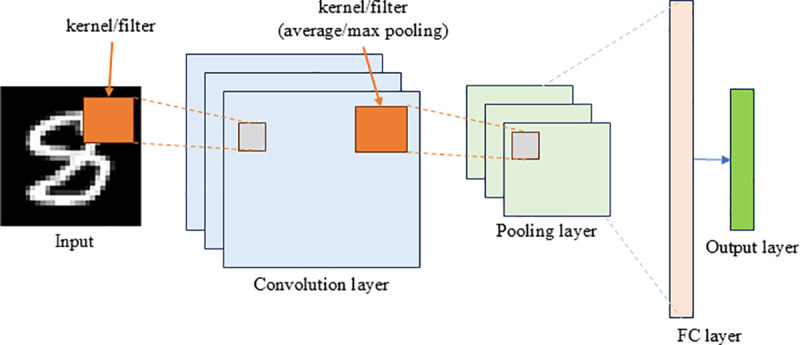
Typical CNN architecture.

Image feature extraction aims to transform raw image data into numerical features while keeping the essential information. These features are necessary for various downstream tasks, such as image classification and semantic segmentation. As one of the deep learning neural network architectures used in computer vision, CNN is often used for multi-source image extraction because it has demonstrated effective and efficient performance in image processing tasks [65]. As is shown in Fig 2, CNN has three layers [66]: (1) a convolutional layer that learns feature representations of the inputs; (2) a pooling layer that seeks to reduce the image size while preserving important characteristics using average pooling or max pooling; and (3) a fully connected (FC) layer that performs high-level reasoning and is connected to the output layer (e.g., softmax layer for classification tasks). Kernels or filters are small matrices that perform convolution operations on the input data.

For street view images, the CNN-based semantic segmentation models are often used to analyze and classify the image pixels into different object categories. Common semantic segmentation models include PSPNet [67], Place365 [68], FCN-8s [69], SegNet [70], DeepLabv3 [71], and DeepLabv3+ [72]. The elements extracted from images and used for housing price prediction include buildings, vegetation, sky, sidewalks, pedestrians, etc. For housing photos and remote sensing images, CNN is applied for image classification by using the images as inputs and predetermined labels as outputs, such as housing price levels (cheap/expensive) and view types (urban/suburban areas). Researchers first train a CNN model with minor manually labelled images and then apply the pre-trained model to classify remaining images with transfer learning and fine-tuning. Some studies use the feature vectors of CNN’s specific layers as input features, which are combined with other numerical feature vectors to formulate the final feature space [57,73].

## Overall research methodology

**Fig 3:**
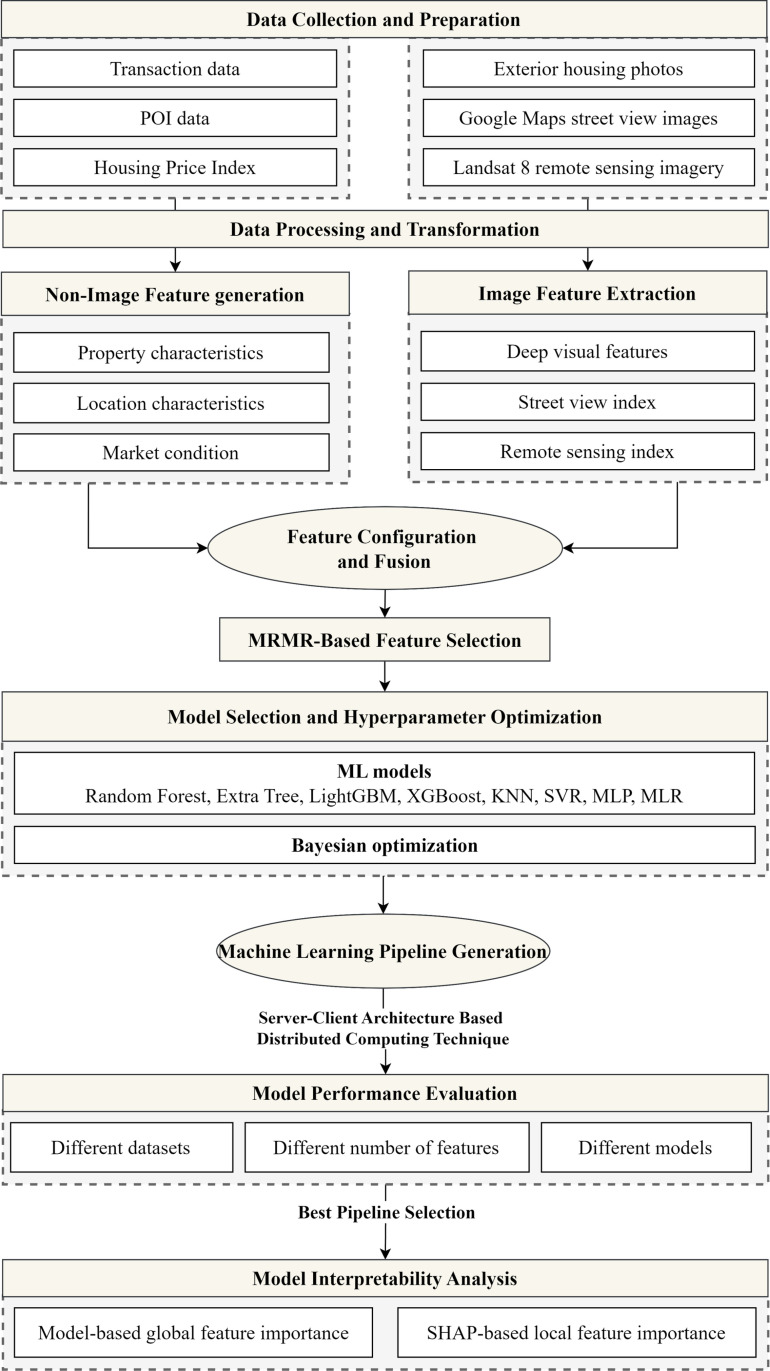
Overall research methodology of this study.

Fig 3 shows our proposed ML framework. The process starts with collecting non-image data and image data. After data processing and transformation, feature generation and extraction are applied to identify the non-image and image features, followed by feature configuration and fusion. The minimum redundancy maximum relevance (MRMR) is used for feature selection. Bayesian optimization is applied to optimize the hyperparameters of eight ML models. A series of ML pipelines are generated by considering different feature configuration parameters, the number of selected features by MRMR, and ML models. The pipelines are then executed using a server-client architecture-based distributed computing technique. The model performances are evaluated using six criteria and statistical tests. The best-performing ML pipeline is selected and used for model interpretability analysis regarding model-based global feature importance and SHAP-based local feature importance.

## Study area and data

### Study area

There are three regions (Hong Kong Island, Kowloon, and New Territories) with 18 districts altogether in Hong Kong. Private apartments sold in the secondary residential market of Hong Kong are selected due to (1) large valuation demands. Private apartments are the most common property type in the open market of Hong Kong. More than 60% of the yearly transaction volume is from the secondary residential market between 2020 and 2022 [74]. (2) sufficient transaction data. There are many mature real estate agencies in Hong Kong, such as Centaline Property, Midland Realty, 28Hse, etc. They provide detailed transaction data lists for individual apartments on their websites. (3) abundant geoinformation database. Hong Kong government has developed an online platform called Common Spatial Data Infrastructure (CSDI) to provide different spatial data types.

### Data collection and preparation

The multi-source datasets in this study consist of (1) non-image data: transaction data, POI data, and housing price index; and (2) image data: exterior housing photos, Google Maps Street view images, and Landsat 8 remote sensing imagery. Before collecting the data, we double-checked the terms and conditions for the data source to ensure the data collection and analysis methods fully meet the protocols.

#### Transaction data, POI data, and housing price index.

**Fig 4:**
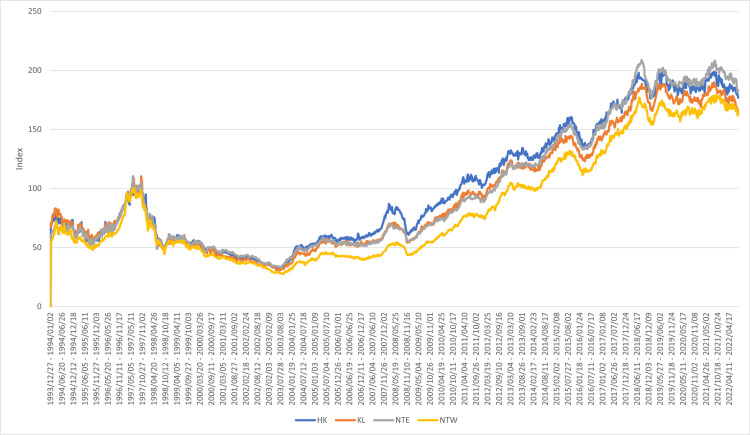
CCL index in Hong Kong.


A web crawler via Python was developed to collect the apartment transaction records from 28hse.com. The data attributes include transaction date, the district where the apartment is located, estate name, unit address, floor level, gross floor area (GFA) of the apartment, and the transaction price. A total of 26,377 transaction records from July 2021 to December 2021 were collected. POI data in Hong Kong were collected from the CSDI platform. Centa-city leading (CCL) index reflects the housing price fluctuations in Hong Kong (Fig 4). It is a weekly index generated by Centaline Property using the transaction prices of properties with large transaction volumes in Hong Kong. CCL index is expressed as:

$$\begin{array}{c} \begin{array}{c} I_{t}=\frac{V_{t}\;}{V_{t-1}}\times I_{t-1} \end{array} \end{array}$$ (1)

where $I_{t}$ is the CCL in week t; $V_{t}$ is the total market value of representative properties in week t.

**Fig 5:**
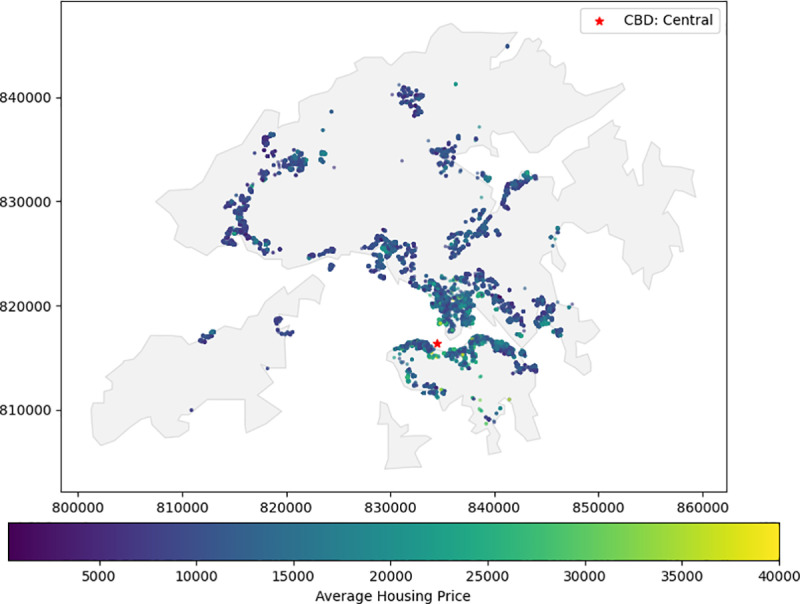
Spatial distribution of average housing prices in Hong Kong.

We first identified the columns related to the transaction date, property address, property characteristics, and transaction price. Other columns were discarded. Data cleaning was applied to address the inconsistent and missing values and outliers. Then, the CCL index was inserted into the transaction records by matching the transaction date with the index’s release date. Lastly, the property address was formulated as a string to obtain geographic coordinates using the CSDI platform’s geocoding API. A total of 22,888 transaction records with the CCL index and geographic coordinates were finalized. The pseudo-code is provided in Algorithm 1. The spatial distribution of average housing prices is provided in Fig 5. The red marker refers to Central, the central business district (CBD) of Hong Kong. The housing prices near the CBD area are relatively higher than in other regions.

Algorithm 1 Data preparation procedures for raw transaction records

Input: Raw data transaction records $D_{r}$; CCL index data T

Output: Clean data transaction records $D_{c}\;$ with CCL index and geographic coordinates

/*Data cleaning*/

1: Identify the columns related to the transaction date, property addresses, property characteristics, and transaction price. Other columns are discarded.

2: Delete data rows with missing values /*3207 samples are removed*/

3: Remove the string of units in the GFA column (i.e., ft2)

/*Outliers are defined as data samples that are not within the interval: [Q1 - 1.5 * (Q3 - Q1), Q3 + 1.5 * (Q3 -Q1), Q1 is 25th percentile and Q3 is 75th percentile]

4: Use boxplot to detect outliers in the GFA column and delete rows with outliers. /* 122 samples are removed*/

5: Unify the units in the transaction price column /* (i.e., Million HK$ to HK$)*/

6: Use boxplot to detect outliers in the transaction price column and delete rows with outliers /*160 samples are removed*/

/*Assign CCL index based on the transaction date*/

7: Identify the time string format in the transaction date column. /*i.e., year-month-day*/

8: Extract the year, month, and day. Assign each record the latest CCL index before the transaction date from T.

/*Get geographic coordinates based on property address*/

9: Create a new column of property address string that includes district name, estate name, and building block name (if any).

10: Use the location search API of the CSDI platform to derive the HK1980 Grid (northing, easting) and the WGS84 (latitude, longitude) coordinates based on the address string.

#### Exterior housing photos.

A web crawler was designed to collect the exterior images of all estates from 28hse.com, and then we matched them with transaction records using the unique estate ID. A total of 2,372 estate photos were finalized.

#### Google Maps street view images.

We first downloaded the road centerline network from the CSDI Platform of Hong Kong. Then, we imported the clean transaction records and the road centerline network into ArcGIS Pro to sample the image collection points at 50 m intervals. A total of 46,147 sampling points within 1 km of the properties were kept and exported to an Excel file with point ID, latitude, and longitude columns. Google Street View API was used to identify the pano_id parameter of each sampling point to collect panoramic street view images. The image tiles were collected based on the pano_id and stitched into a full-view panoramic image with a size of 1664x832 pixels. For sampling points without available panoramic images, we turned to collect the single-view images with four different headings: front view (heading=0), right view (heading=90), rear view (heading=180) and left view (heading=270). The *FOV* parameter was set to 90 degrees, and the *pitch* parameter was set to 0. The image size was set to 640x360 pixels, and the collection date was set to July 2021. A total of 42,398 panoramic images and 13,324 (3331x4) single-view images were collected, and 418 sampling points were discarded because there were no available street view images in these points.

#### Landsat 8 remote sensing imagery.

The Earth Explorer of the United States Geological Survey was used to collect Landsat 8-based GeoTIFF files. We first identified the search criteria by drawing the polygon around the target area (Hong Kong), selecting the Landsat 8 data set, and determining the range of data generation date as 2021/07/01 to 2021/12/31. Among the forty-six search results, we chose one GeoTIFF file generated on 2021/12/05, which covers all housing transaction samples and has the best image quality without cloud interference.

## Feature engineering

### Non-image feature generation

Feature generation creates new features from the primary feature space or raw data to increase the robustness and generalizability of the model [75]. We identify numerical feature spaces as property characteristics, location characteristics, and market conditions. Property characteristics include floor level and floor area. Location characteristics include geographic coordinates, Wi-Fi hotspot density, POI density, POI diversity, and POI accessibility. The POI database has 8 POI categories, 18 POI classes, over 100 POI types, and over 38,000 geocoded POI places in Hong Kong (Table 2). POI density is identified as the number of POI places within 1 km of the property. POI diversity includes the number of POI classes and types within 1 km of the property, the entropy-based diversity index of POI class and types within 1 km of the property. The entropy-based diversity index is expressed as:

**Table 2:** POI database in Hong Kong.

POI Categories	POI Class	No. of POI types	No. of POI places			
**Hong Kong**	**Kowloon**	**New Territory**	**Total**
Community Facilities	Commercial Facility (CMF)	7	677	1031	1327	3035
	Community Facility (COM)	4	86	144	424	654
	Cultural Facility (CUF)	11	271	216	678	1165
	Municipal Facility (MUF)	7	564	694	2958	4216
	**Total**	29	1598	2085	5387	9070
Education	Educational (SCH)	9	707	1152	1973	3832
Recreation	Aquatics (AQU)	2	18	0	59	77
	Conservation (PAK)	7	8	0	48	56
	Leisure Facility (RSF)	21	1382	1859	4667	7908
	Tourism and Hiking (TRH)	3	45	10	80	135
	**Total**	33	1453	1869	4854	8176
Medical	Health Care (HNC)	5	251	369	479	1099
Public Service/Facilities	Burial Ground (BGD)	4	58	26	102	186
	Public Service (GOV)	29	666	531	947	2144
	Utilities/Installation (UTI)	5	518	545	1911	2974
	**Total**	38	1242	1102	2960	5304
Religion	Religious (REM)	7	281	548	864	1693
Transportation	Bus Stop (BUS)	2	256	352	767	1375
	Transportation Furniture (TRF)	5	128	170	627	925
	Transportation (TRS)	16	528	867	1660	3055
	**Total**	30	1193	1937	3918	7048
Tourism	Accommodation (AMD)	4	268	1321	276	1865
	**Total**	148	6712	9835	19847	36394


$$\begin{array}{c} \begin{array}{c} D_{x,class}=-\underset{i=1}{\overset{n}{\sum }}\;\frac{N_{i}}{N_{total}}\log_{2}\left(\frac{N_{i}}{N_{total}}\right)\;\; \end{array} \end{array}$$ (2)

$$\begin{array}{c} \begin{array}{c} D_{x,type}=-\underset{j=1}{\overset{m}{\sum }}\;\frac{N_{j}}{N_{total}}\log_{2}\left(\frac{N_{j}}{N_{total}}\right) \end{array} \end{array}$$ (3)

where $D_{x,class}$ and $D_{x,type}$ are the diversity index of POI classes and types for property x. m and n are total number of POI classes and types. $N_{i}$ and $N_{j}$ are the number of POI places that belong to class i and type j within 1 km of the property x. $N_{total}$ is the total number of POI places within 1 km of the property x.

For POI accessibility, we identified 12 POI types according to the previous research results regarding the influencing factors of housing prices, including mall/shopping centre/commercial complex (MAL) and supermarket (SMK) [76], kindergarten (KDG), primary school (PRS) and secondary school (SES) [77,78], park (PAR), playground (PLG) and minor open space (RGD) [79,80], bus terminus (BUS), green minibus terminus (MIN) and railway station entrance (MTA) [81], and car park (CPO) [82]. POI accessibility is represented with a binary variable indicating whether a POI is within a predetermined circular distance range of a property. Market condition is proxied using the latest CCL index before the transaction date.

### Image feature extraction

In this study, three types of image features are extracted from exterior housing photos, street view and remote sensing images, including deep visual features after dimension reduction, street view and remote sensing index. Detailed procedures are introduced in the following three subsections.

#### Deep visual features after dimension reduction.

CNN is used to extract deep visual features because it leverages local receptive fields, shows computational effectiveness with sharing parameters, and efficiently and effectively captures the spatial and structural information in images [83]. Specifically, CNN is first used to extract the feature vectors of the last FC layer before the output layer. As the FC layer is usually high-dimensional, directly integrating the high-dimensional features will cause the curse of dimensionality, which can increase computational complexity and degrade model performance. Therefore, a nonlinear t-distributed stochastic neighbor embedding (t-SNE) is then used to reduce the FC layer’s dimension into low-dimensional space to avoid the curse of dimensionality, retain the most relevant features and achieve higher computational efficiency. The t-SNE is an unsupervised nonlinear dimensionality reduction technique that visualizes high-dimensional data by giving each data point a location in a two- or three-dimensional space [84]. The deep visual features after dimension reduction are extracted from exterior housing photos, and each property is attached with corresponding photo-based features.

#### Semantic segmentation-based street view index.

A review study benchmarks the semantic segmentation models, and DeepLabv3+ performs best on the Cityscapes and PASCAL VOC datasets [85]. The two public datasets are widely used for benchmarking in computer vision tasks such as object detection, semantic segmentation, and classification. As the Cityscape dataset is related to the semantic understanding of urban street scenes, the DeepLabv3+ model pre-trained on the Cityscapes dataset was used to extract the view index from street view images. Three types of view index are used in this study, including the building view index (BVI), sky view index (SVI), and vegetation view index (VVI). These three indices have demonstrated significant impacts on housing prices, and the formulas are expressed as [53]:


$$\begin{array}{c} \begin{array}{c} v_{c}=\frac{p_{c}}{p_{t}},c\in building,sky,vegetation \end{array} \end{array}$$ (4)

where $v_{c}$ is the percentage of visual element class c in the SVI. $p_{c}$ is the total number of pixels associated with the visual element class c. $p_{t}$ is the total number of pixels in the street view image. A property’s view index is represented by averaging the view index of sampling points within a predetermined radius of the property.

#### Remote sensing index.

Landsat 8 has eleven bands, namely, different ranges of frequencies along the electromagnetic spectrum. Band 3 (green), band 4 (red), band 5 (near infrared, NIR), and band 6 (shortwave infrared 1, SWIR 1) of remote sensing images were used to calculate three types of remote sensing index, namely, the Normalized Difference Vegetation Index (NDVI), the Normalized Difference Water Index (NDWI), and the Normalized Difference Built-up Index (NDBI). The formulas are expressed as follows:


$$\begin{array}{c} \begin{array}{c} NDVI=\frac{Band\;5-Band\;4}{Band\;5+Band\;4} \end{array} \end{array}$$ (5)

$$\begin{array}{c} \begin{array}{c} NDWI=\frac{Band\;3-Band\;6}{Band\;3+Band\;6} \end{array} \end{array}$$ (6)

$$\begin{array}{c} \begin{array}{c} NDBI=\frac{Band\;6-Band\;5}{Band\;6+Band\;5} \end{array} \end{array}$$ (7)

Similar to view index, a property’s remote sensing index is represented by averaging the index within a predetermined radius of the property.

### Feature configuration and fusion

**Fig 6:**
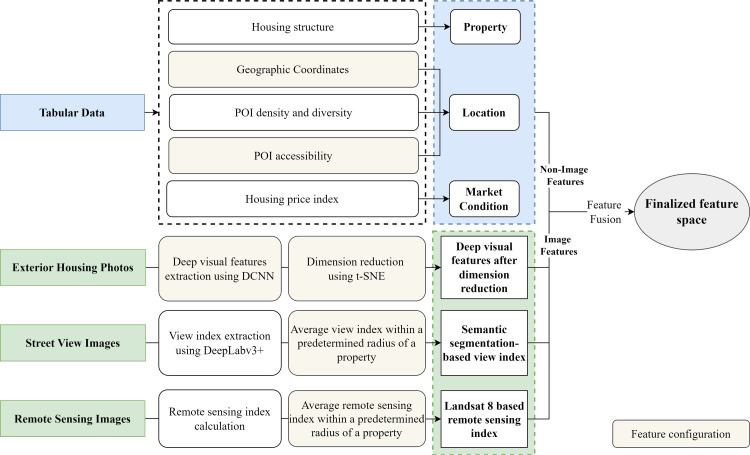
Feature configuration and fusion framework.

Fig 6 shows the proposed feature configuration and fusion framework. First, feature configuration is applied to several features by selecting different feature configuration parameters to create various feature combinations. The features to be configured include geographic coordinates, POI accessibility, deep visual features with DCNN and after dimension reduction, average view and remote sensing index. Non-image features extracted from tabular data include property, location and market condition. Image features extracted from exterior housing photos, street view images, and remote sensing images correspond to deep visual features after dimension reduction, semantic segmentation-based view index, and Landsat 8-based remote sensing index. The non-image and image features are then concatenated and fused into the finalized feature space.

#### Feature configuration.

Table 3 presents the feature configuration parameter descriptions and candidates. The geographic coordinates can be represented by either easting and northing in Hong Kong 1980 grid system or latitude and longitude in The World Geodetic System 1984 (WGS84). Four DCNN types are tested to extract deep visual features from housing photos, including GoogleNet [86], AlexNet [65], VGG16 [87], and ResNet-101 [88]. The number of reduced dimensions using t-SNE is set as either 2-dimensional or 3-dimensional. Two distance radiuses are used to calculate POI accessibility, i.e., 300 and 500 m; Two distance radiuses are used to derive view index and remote sensing index, i.e., 500 and 1000 m. A total of 128 feature combinations are generated after feature configuration.

**Table 3:** Feature configuration parameters.

Parameter descriptions	Parameter candidates
Geographic coordinate types	[Easting and Northing, Longitude and Latitude]
Distance radius used to calculate POI accessibility	[300 m, 500 m]
DCNN types used for deep visual feature extraction	[GoogleNet, AlexNet, VGG16, ResNet-101]
Number of reduced dimensions using t-SNE	[2-dimensional, 3-dimensional]
Distance radius used to calculate view index	[500 m, 1000 m]
Distance radius used to calculate remote sensing index	[500 m, 1000 m]

#### Non-image and image feature fusion.

Tabular data is used to generate 23 non-image features that are related to property, location, and market conditions. Exterior housing photos are processed with DCNN and t-SNE to generate two or three features after 2D or 3D t-SNE. Street view images are segmented with a pre-trained DeepLabv3+ model to generate three features related to the view of building, sky and vegetation. Remote sensing images are processed to produce three features related to the NDVI, NDWI and NDBI. The non-image and image features are concatenated and fused to formulate the finalized feature space with 31 or 32 features. The feature space containing all potential features is summarized in Table 4, where the last two columns show each feature’s mean value and standard deviation (SD).

**Table 4:** Feature space with all potential features.

Feature category	Feature name	Descriptions	Mean	SD
Dependent variable	Price	Average transaction price of a property (HK$/sqft)	13150.5	5362.76
**Non-Image Features**				
**Property**				
Housing structure	Floor	The floor number of a property	2.97	2.299
	Area	Gross floor area of property	666.89	295.119
**Location**				
Geographic Coordinates	x	Easting of a property (HK 1980 Grid coordinate system)	832735.19	9098.315
	y	Northing of a property (HK 1980 Grid coordinate system)	823595.59	6896.842
	Longitude	Longitude of a property (World Geodetic System 1984)	114.14	0.088
	Latitude	Latitude of a property (World Geodetic System 1984)	22.35	0.062
Wi-Fi density	wifi_hk	Number of Wi-Fi hotspots within 1km radius of the property.	85.18	65.759
POI density	POI_density	Number of POI places within 1 km radius of the property	470.78	289.023
POI diversity	Num_class	Number of POI classes within 1 km radius of the property	14.43	1.106
	Num_types	Number of POI types within 1 km radius of the property	55.71	15.129
	Class_diversity	Entropy-based diversity index of POI classes within 1 km radius of a property	3.33	0.187
	Type_diversity	Entropy-based diversity index of POI types within 1 km radius of a property	4.81	0.375
POI accessibility	MAL_Walk300	Binary variable: whether a MAL type POI exists within 300m radius of a property	0.71	0.452
	MAL_Walk500	Binary variable: whether a MAL type POI exists within 500m radius of a property	0.87	0.333
	SMK_Walk300	Binary variable: whether a SMK type POI exists within 300m radius of a property	0.83	0.373
	SMK_Walk500	Binary variable: whether a SMK type POI exists within 500m radius of a property	0.93	0.250
	KDG_Walk300	Binary variable: whether a KDG type POI exists within 300m radius of a property	0.83	0.372
	KDG_Walk500	Binary variable: whether a KDG type POI exists within 500m radius of a property	0.95	0.221
	PRS_Walk300	Binary variable: whether a PRS type POI exists within 300m radius of a property	0.66	0.474
	PRS_Walk500	Binary variable: whether a PRS type POI exists within 500m radius of a property	0.88	0.323
	SES_Walk300	Binary variable: whether a SES type POI exists within 300m radius of a property	0.55	0.497
	SES_Walk500	Binary variable: whether a SES type POI exists within 500m radius of a property	0.79	0.409
	PAR_Walk300	Binary variable: whether a PAR type POI exists within 300m radius of a property	0.28	0.447
	PAR_Walk500	Binary variable: whether a PAR type POI exists within 500m radius of a property	0.55	0.497
	PLG_Walk300	Binary variable: whether a PLG type POI exists within 300m radius of a property	0.53	0.499
	PLG_Walk500	Binary variable: whether a PLG type POI exists within 500m radius of a property	0.79	0.410
	RGD_Walk300	Binary variable: whether a RGD type POI exists within 300m radius of a property	0.72	0.447
	RGD_Walk500	Binary variable: whether a RGD type POI exists within 500m radius of a property	0.87	0.332
	BUS_Walk300	Binary variable: whether a BUS type POI exists within 300m radius of a property	0.67	0.470
	BUS_Walk500	Binary variable: whether a BUS type POI exists within 500m radius of a property	0.91	0.287
	MIN_Walk300	Binary variable: whether a MIN type POI exists within 300m radius of a property	0.59	0.492
	MIN_Walk500	Binary variable: whether a MIN type POI exists within 500m radius of a property	0.82	0.382
	MTA_Walk300	Binary variable: whether a MTA type POI exists within 300m radius of a property	0.38	0.486
	MTA_Walk500	Binary variable: whether a MTA type POI exists within 500m radius of a property	0.59	0.492
	CPO_Walk300	Binary variable: whether a CPO type POI exists within 300m radius of a property	0.91	0.279
	CPO_Walk500	Binary variable: whether a CPO type POI exists within 500m radius of a property	0.97	0.180
**Market Condition**				
Housing rice index	CCL	The latest CCL index before the transaction date of a property	187.99	10.795
**Image Features**				
Feature vectors	GoogleNet_2d1	The first value of embedded vectors applying 2-d t-SNE to FC layer of GoogleNet	0.50	0.187
	GoogleNet_2d2	The second value of embedded vectors applying 2-d t-SNE to FC layer of GoogleNet	0.49	0.210
	GoogleNet_3d1	The first value of embedded vectors applying 3-d t-SNE to FC layer of GoogleNet	0.51	0.193
	GoogleNet_3d2	The second value of embedded vectors applying 3-d t-SNE to FC layer of GoogleNet	0.50	0.198
	GoogleNet_3d3	The third value of embedded vectors applying 3-d t-SNE to FC layer of GoogleNet	0.48	0.196
	AlexNet_2d1	The first value of embedded vectors applying 2-d t-SNE to FC layer of AlexNet	0.51	0.201
	AlexNet_2d2	The second value of embedded vectors applying 2-d t-SNE to FC layer of AlexNet	0.51	0.222
	AlexNet_3d1	The first value of embedded vectors applying 3-d t-SNE to FC layer of AlexNet	0.54	0.211
	AlexNet_3d2	The second value of embedded vectors applying 3-d t-SNE to FC layer of AlexNet	0.52	0.198
	AlexNet_3d3	The third value of embedded vectors applying 3-d t-SNE to FC layer of AlexNet	0.47	0.191
	VGG16_2d1	The first value of embedded vectors applying 2-d t-SNE to FC layer of VGG16	0.51	0.215
	VGG16_2d2	The second value of embedded vectors applying 2-d t-SNE to FC layer of VGG16	0.48	0.221
	VGG16_3d1	The first value of embedded vectors applying 3-d t-SNE to FC layer of VGG16	0.50	0.203
	VGG16_3d2	The second value of embedded vectors applying 3-d t-SNE to FC layer of VGG16	0.50	0.206
	VGG16_3d3	The third value of embedded vectors applying 3-d t-SNE to FC layer of VGG16	0.50	0.191
	ResNet101_2d1	The first value of embedded vectors applying 2-d t-SNE to FC layer of ResNet101	0.51	0.239
	ResNet101_2d2	The second value of embedded vectors applying 2-d t-SNE to FC layer of ResNet101	0.53	0.225
	ResNet101_3d1	The first value of embedded vectors applying 3-d t-SNE to FC layer of ResNet101	0.50	0.227
	ResNet101_3d1	The second value of embedded vectors applying 3-d t-SNE to FC layer of ResNet101	0.51	0.213
	ResNet101_3d1	The third value of embedded vectors applying 3-d t-SNE to FC layer of ResNet101	0.47	0.187
View index	building500	Average building view index within 500m radius of a property	0.21	0.086
	building1000	Average building view index within 1000m radius of a property	0.20	0.073
	sky500	Average sky view index within 500m radius of a property	0.29	0.054
	sky1000	Average sky view index within 1000m radius of a property	0.30	0.048
	vegetation500	Average vegetation view index within 500m radius of a property	0.14	0.062
	vegetation1000	Average vegetation view index within 1000m radius of a property	0.14	0.051
RS index	NDVI500	Average NDVI within 500m radius of a property	0.44	0.065
	NDVI1000	Average NDVI within 1000m radius of a property	0.46	0.066
	NDWI500	Average NDWI within 500m radius of a property	0.51	0.063
	NDWI1000	Average NDWI within 1000m radius of a property	0.50	0.067
	NDBI500	Average NDBI within 500m radius of a property	0.40	0.032
	NDBI1000	Average NDBI within 1000m radius of a property	0.39	0.031

### MRMR-based feature selection

Feature selection aims to build a feature subset based on the original feature set to reduce effects from data noise or irrelevant variables and still provide good prediction results [89]. This study uses the MRMR method to select the most efficient and distinctive features. MRMR uses mutual information to find the optimal feature set by minimizing the redundancy between selected features and maximizing the relevance between selected features and target variable simultaneously [90] expressed as:


$$\begin{array}{c} \max\;\;\;\Phi \;\;\;\left(S\right)=D-R=\frac{1}{\left|S\right|}\underset{x_{i}\in S}{\sum }\;I\left(x_{i},y\right)-\frac{1}{{\left|S\right|}^{2}}\underset{x_{i},x_{j}\in S}{\sum }\;I\left(x_{i},x_{j}\right) \end{array}$$ (8)

where D is the relevance between the selected feature set S and target variable y. R is the redundancy between the selected features in S. I(·) is the mutual information operator. The procedures of the MRMR algorithm are shown in Algorithm 2.

Algorithm 2 Minimum Redundancy Maximum Relevance

Input: Dataset D, feature set ℱ, number of selected features q

Output: A feature subset $\left\{f_{1},f_{2},\ldots ,f_{q}\right\}\subseteq ℱ$

1: S=∅

2: for k=1,2,…,q do

3: $f_{k}\in argmax\{\;\;\;\Phi \;\;\;\left(f\right)|f\in ℱ/S\}$

4: Add $f_{k}$ to S

5: end for

6: return S

### Model selection and hyperparameter optimization

Model generation aims to choose a learning algorithm automatically and simultaneously set its hyperparameters to optimize model performance [91]. Previous studies on residential property valuation have shown that tree-based ML models perform the best [16,92,93]. Therefore, four tree-based ML models were selected: Random Forest, Extra Tree, XGBoost, and LightGBM.

Random Forest (RF) builds a new dataset with a replacement (bootstrap sampling) from an existing dataset and trains several decision tree models with randomly selected features on the new dataset [94]. The predictions of each decision tree model are aggregated into the final prediction. Extra Trees (ET) splits nodes by choosing cut points fully at random and uses the whole learning sample (rather than a bootstrap replica) to grow the trees [95]. XGBoost is a gradient boosting tree (GBDT) based algorithm that constructs the objective function of model deviation and regularization term to prevent over-fitting [96]. LightGBM is also a GBDT base algorithm that proposes two novel techniques, i.e., gradient-based one-side sampling (GOSS) and exclusive feature bundling (EFB), to realize faster training efficiency and lower memory usage. Four non-tree-based models are also included for benchmark comparison, including k-nearest neighbours (KNN), support vector regression (SVR), multiple layer perceptron (MLP), and multiple linear regression (MLR).

Bayesian optimization (BO) is an iterative stochastic optimization framework for the CASH problem [97]. It first builds a probabilistic surrogate model (Gaussian process or tree-based model) mapping from the hyperparameters to the objective metrics. Then, it defines an acquisition function to decide which hyperparameter configuration to evaluate next, balancing the exploration and exploitation during the search process [98]. The hyperparameter search spaces of tree-based and benchmark models are presented in Table 5.

**Table 5:** Hyperparameter search spaces.

Algorithm	Parameter	Value
Random Forest	n_estimators	Integer uniform: [10, 200]
	min_samples_split	Integer uniform: [2,21]
	min_samples_leaf	Integer uniform: [1,21]
Extra Tree	n_estimators	Integer uniform: [10, 200]
	min_samples_split	Integer uniform: [2,21]
	min_samples_leaf	Integer uniform: [2,21]
	bootstrap	[‘True’, ‘False’]
XGBoost	n_estimators	Integer uniform: [10, 200]
	max_depth	Integer uniform: [1,10]
	min_child_weight	Integer uniform: [1,20]
	subsample	[0.1, 0.2, 0.3, …, 1]
	learning_rate	[0.001, 0.01, 0.1, 0.5]
LightGBM	num_leaves	Integer uniform: [20, 50]
	max_depth	Integer uniform: [1, 10]
	n_estimators	Integer uniform: [10, 200]
	learning_rate	[0.001, 0.01, 0.1, 0.5]
KNN	*n_neighbors*	Integer uniform: [5, 50]
	*weights*	[‘uniform’, ‘distance’]
SVR	*eplison*	[0.001, 0.01, 0.05, 0.1, 0.5, 1]
	*C*	Integer uniform: [1, 100]
MLP	*hidden_layer_sizes*	Integer uniform: [50, 150]
	*learning_rate_init*	[0.0001, 0.001, 0.005, 0.01]

## ML pipeline generation and evaluation

### Pipeline generation and execution

A total of 128 datasets are created with various combined features and split into training and test sets with a ratio of 80:20. As there are 31 features in total, and the number of selected features using the MRMR method is set as 1, 6, 11, 16, 21, 26, 31. Each dataset with selected features is trained with four ML models. A total of pipelines are generated and executed by server-client based distributed computing technique.

The distributed computing technique runs programs across several computers on a network to achieve high-performance scientific computing. The server-client architecture requires that the server divides the tasks of ML pipeline execution to the clients, which run the tasks and send the pipeline performance results back to the server. The experiments have been conducted by selecting one laptop as a server and three desktops as clients. A 10-core Intel i7-12650H processor (2.30 GHz) with 16 GB RAM is used as the server that sends the pipeline training tasks to: (1) desktop client 1: an 8-core AMD Ryzen 7 5700X processor (3.40 GHz) with 32GB RAM; (2) desktop client 2: a 6-core Intel i5-10500 processor (3.10 GHz) with 16GB RAM; and (3) desktop client 3: a 4-core Intel i7-4770 processor (3.40 GHz) with 32GB RAM. The network condition is configured as Wi-Fi with 1000Mbps uplink bandwidth and 1000Mbps downlink bandwidth. The proposed machine learning and distributing computing experiments are implemented in Python3 using *scikit-learn* and *multiprocessing* package.

### Pipeline evaluation and interpretation

#### Evaluation criteria.

The pipelines are measured with multiple evaluation criteria to identify the best performing one: root mean squared error (RMSE) (Eq. 9), percentage of RMSE (Eq. 10), mean absolute error (MAE) (Eq. 11), percentage of MAE (Eq. 12), R squared (Eq. 13), and coefficient of dispersion (COD) (Eq. 14).


$$\begin{array}{c} RMSE=\sqrt{\frac{1}{n}\underset{i=1}{\overset{n}{\sum }}\;{\left(y_{i}-{\hat{y}}_{i}\right)}^{2}} \end{array}$$ (9)

$$\begin{array}{c} \begin{array}{c} RMSE\left(\%\right)=\frac{\sqrt{\frac{1}{n}\sum_{i=1}^{n}{\left(y_{i}-{\hat{y}}_{i}\right)}^{2}}}{\frac{1}{n}\sum_{i=1}^{n}\left|y_{i}\right|}\times 100 \end{array} \end{array}$$ (10)

$$\begin{array}{c} \begin{array}{c} MAE=\frac{1}{n}\underset{i=1}{\overset{n}{\sum }}\;\left|y_{i}-{\hat{y}}_{i}\right| \end{array} \end{array}$$ (11)

$$\begin{array}{c} \begin{array}{c} MAE\left(\%\right)=\frac{\sum_{i=1}^{n}\left|y_{i}-{\hat{y}}_{i}\right|}{\sum_{i=1}^{n}\left|y_{i}\right|}\times 100 \end{array} \end{array}$$ (12)

$$\begin{array}{c} \begin{array}{c} R^{2}=1-\frac{\sum_{i=1}^{n}{\left(y_{i}-{\hat{y}}_{i}\right)}^{2}}{\sum_{i=1}^{n}{\left(y_{i}-\bar{y}\right)}^{2}},\bar{y}=\frac{1}{n}\underset{i=1}{\overset{n}{\sum }}\;y_{i} \end{array} \end{array}$$ (13)

$$\begin{array}{c} \begin{array}{c} COD=\frac{\frac{100\%}{n}\sum_{i=1}^{n}\left|r_{i}-\tilde{r}\right|}{\tilde{r}} \end{array} \end{array}$$ (14)

where $y_{i}$ = the actual value, ${\hat{y}}_{i}$ = the predicted value, $\bar{y}$ = the mean of the actual values, COD = coefficient of dispersion, $r_{i}={\hat{y}}_{i}/y_{i}$, and $\tilde{r}$ = the median of $r_{i}$ in a dataset with n numbers of samples.

#### Statistical evaluation.

The statistical evaluation framework includes three steps: (1) calculate the performances of different pipelines according to the six evaluation criteria to obtain average rankings for each pipeline; (2) use the Wilcoxon signed-rank test to test whether two groups of pipelines perform equally; (3) use Friedman test to test whether multiple groups of pipelines perform equally.

The pipeline performances are first evaluated using the six evaluation criteria. $R_{ij}$ refers to the ranking of the ith pipeline using the jth evaluation criterion. A pipeline’s ranking $R_{i}$ is calculated by averaging $R_{ij}$:


$$\begin{array}{c} \begin{array}{c} R_{i}=\frac{1}{J}\underset{j}{\overset{J}{\sum }}\;R_{ij},i=1,2,3,..,I \end{array} \end{array}$$ (15)

Where I and J are the total number of pipelines and evaluation criteria, respectively. The Wilcoxon signed-rank test is a non-parametric alternative of the paired t-test [99], and it aims to perform paired comparisons and test whether one pipeline group (e.g., pipelines using Extra Tree) performs significantly better than the other one (e.g., pipelines using Random Forest). The Friedman test is also a non-parametric statistical test method [100], and it is used to test whether there are statistically significant performance differences among multiple pipeline groups (e.g., pipelines using different datasets).

#### Model interpretation.

The optimal pipeline will be interpreted by the SHAP method, which explains the prediction of an instance by computing the contribution of each feature to the prediction by computing Shapley values from coalitional game theory [101]. The SHAP value for feature j of observation x, $\phi_{j}\left(x\right)$, is defined as:


$$\begin{array}{c} \phi_{j}\left(x\right)=\underset{S\subseteq \left\{1,2,\ldots ,M\right\}\backslash \left\{j\right\}}{\sum }\;\frac{\left|S\right|!\left(M-\left|S\right|-1\right)!}{M!}\left[f\left(x_{S}\cup \left\{j\right\}\right)-f\left(x_{S}\right)\right] \end{array}$$ (16)

where S is a subset of the features with feature j excluded. M is the total number of features. f is the model’s prediction function.

## Results and discussions

A total of 7168 ML pipelines are generated and trained with the distributed computing technique. After all pipelines are trained, a leaderboard of their performances will be created, including dataset ID, pipeline ID, ML model with optimized hyperparameters, selected features, model performances and corresponding average rankings.

### Model performance analysis

#### Model performances using different datasets.

**Fig 7:**
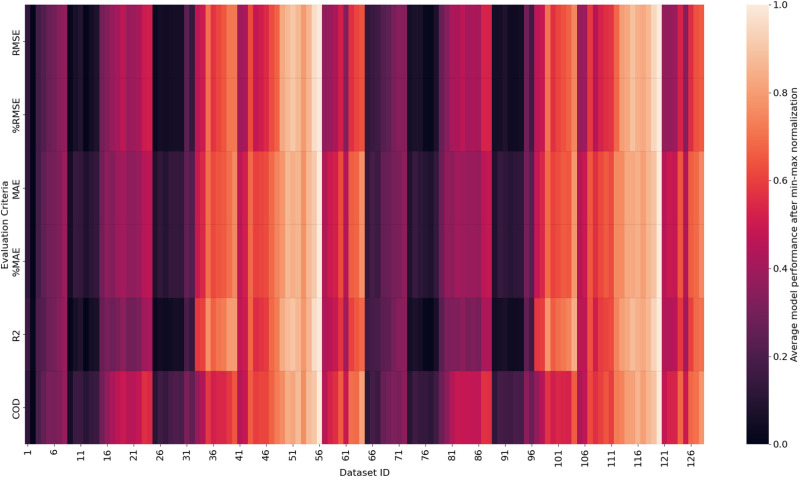
Model performance of different datasets.

Fig 7 illustrates the normalized average model performance of all 128 datasets with six evaluation criteria, and the performances vary among different datasets. The Friedman test is used to test whether there are significant differences between the performances of these datasets, and the p-value is smaller than 0.01, indicating the performances of these datasets are significantly different at the 99% confidence level. Most previous studies used only one dataset to train ML models; however, there is no guarantee that the dataset will lead to the best-performing one. For instance, many distance ranges have been used by previous studies to calculate the street view index of a property, and simply following their parameter settings may not work well for our case. Formulating a series of datasets and searching for the optimal feature configuration parameters is necessary to improve the overall model performance.

#### Model performances selecting different number of features.

**Fig 8:**
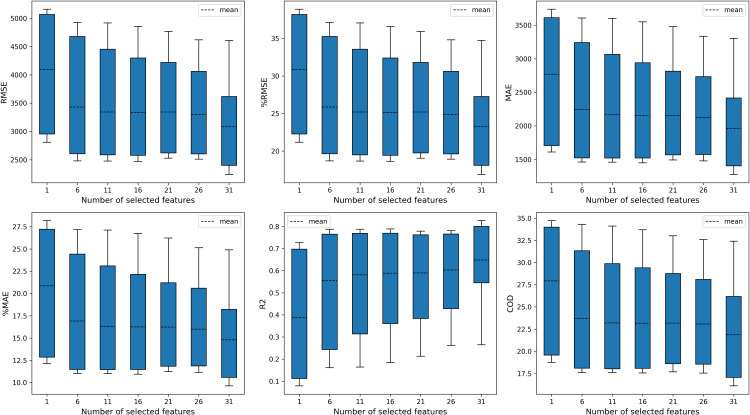
Model performance of different selected features.

Fig 8 presents the mean and distributions of model performances with different numbers of features selected. Selecting more features leads to more accurate and stable prediction results. Table 6 shows the model performance results with varying numbers of features. The Wilcoxon test is used to test whether there are significant differences when selecting different numbers of features, and the model performances with more features selected are statistically better than those with fewer features.

**Table 6:** Model performance results of different number of selected features.

No. of Features	RMSE			%RMSE			MAE			%MAE			R2			COD		
	Min	Mean	Max	Min	Mean	Max	Min	Mean	Max	Min	Mean	Max	Min	Mean	Max	Min	Mean	Max
1	2808.81	4095.11	5159.93	21.17%	30.87%	38.89%	1610.12	2767.91	3737.49	12.14%	20.86%	28.17%	0.079	0.388	0.727	18.73	27.95	34.72
6	2477.91	3431.70	4924.59	18.68%	25.87%	37.12%	1460.97	2243.73	3606.63	11.01%	16.91%	27.19%	0.161	0.556	0.788	17.60	23.72	34.32
11	2475.71	3342.85	4916.11	18.66%	25.20%	37.06%	1460.19	2166.10	3599.30	11.01%	16.33%	27.13%	0.164	0.581	0.788	17.61	23.19	34.13
16	2469.41	3333.68	4856.49	18.61%	25.13%	36.61%	1450.72	2156.72	3549.33	10.93%	16.26%	26.75%	0.184	0.588	0.789	17.55	23.16	33.71
21	2526.99	3343.80	4768.57	19.05%	25.20%	35.94%	1491.44	2153.65	3479.15	11.24%	16.23%	26.22%	0.213	0.590	0.779	17.67	23.18	33.03
26	2509.07	3297.39	4620.28	18.91%	24.85%	34.83%	1478.51	2122.45	3334.57	11.14%	16.00%	25.13%	0.261	0.603	0.782	17.54	23.07	32.59
31	2240.08	3083.39	4607.94	16.88%	23.24%	34.73%	1276.94	1962.66	3303.65	9.62%	14.79%	24.90%	0.265	0.649	0.826	16.09	21.87	32.41

#### Model performances using different models.

**Fig 9:**
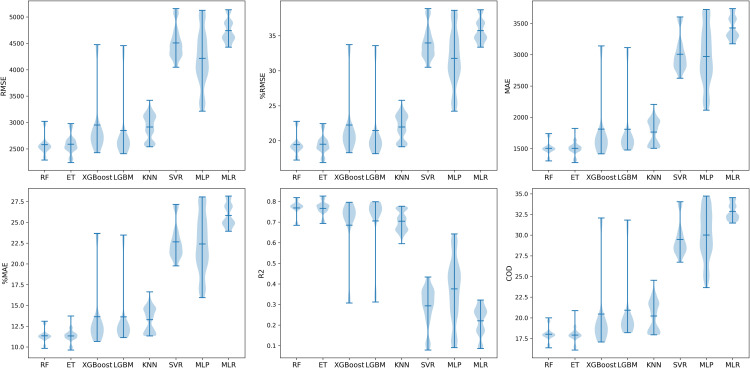
Model performance of different ML models.

Fig 9 presents the model performance distribution of eight ML models using violin plots, which can visualize the distribution of numeric data in a hybrid of box plots and kernel density plots. The extreme values (maximum and minimum) and mean values are shown in blue dashes. Overall, the performances of Random Forest and Extra Tree are more stable than those of other models. The model performance distributions of XGBoost and LGBM are similar with large variance. KNN and MLR perform the best and worst of all the benchmark models, respectively. Table 7 shows the performance results of different ML models. The eight pipeline groups using eight models (RF, ET, XGBoost, LGBM, KNN, SVR, MLP, and MLR) are selected for paired comparisons to find the best-performing model. Each pipeline group has 896 pipelines. The Wilcoxon signed-rank test checks whether one pipeline group performs significantly better than the other. The test results show that Extra Tree performs significantly better than other models among all six evaluation criteria and average rankings.

**Table 7:** Model performance results of different models.

Models	RMSE			%RMSE			MAE			%MAE			R2			COD		
	Min	Mean	Max	Min	Mean	Max	Min	Mean	Max	Min	Mean	Max	Min	Mean	Max	Min	Mean	Max
RF	2287.21	2578.49	3021.76	17.24%	19.44%	22.78%	1303.97	1504.55	1738.89	9.83%	11.34%	13.11%	0.684	0.769	0.819	16.37	18.01	19.99
ET	2240.08	2588.67	2977.66	16.88%	19.51%	22.44%	1276.94	1503.49	1822.37	9.62%	11.33%	13.74%	0.693	0.767	0.826	16.09	17.91	20.87
XGBoost	2428.34	2953.20	4474.77	18.30%	22.26%	33.73%	1415.78	1811.16	3139.87	10.67%	13.65%	23.67%	0.307	0.685	0.796	17.06	20.45	32.08
LGBM	2409.24	2847.29	4458.15	18.16%	21.46%	33.60%	1476.87	1808.42	3114.48	11.13%	13.63%	23.48%	0.312	0.706	0.799	18.20	20.91	31.81
KNN	2539.79	2915.10	3419.43	19.14%	21.97%	25.77%	1504.25	1763.99	2206.32	11.34%	13.30%	16.63%	0.595	0.704	0.777	17.94	20.22	24.53
SVR	4046.56	4508.55	5159.93	30.50%	33.98%	38.89%	2622.70	3006.80	3604.06	19.77%	22.66%	27.17%	0.079	0.293	0.433	26.72	29.47	34.05
MLP	3215.18	4215.06	5127.24	24.23%	31.77%	38.65%	2114.45	2971.65	3723.11	15.94%	22.40%	28.06%	0.090	0.376	0.642	23.65	30.00	34.72
MLR	4428.56	4739.85	5138.46	33.38%	35.73%	38.73%	3175.03	3427.91	3737.49	23.93%	25.84%	28.17%	0.086	0.221	0.321	31.46	32.88	34.54

#### Best pipeline selection.

The pipeline with the highest average ranking is selected as the best; its details are presented in Table 8. The best pipeline selects 31 features and is formulated based on the Extra Tree model. The optimized hyperparameters after BO are also listed in the table. The pipeline performs the best among the six evaluation criteria, with the rankings listed in the bracket. Furthermore, the pipeline is used for model interpretability analysis.

**Table 8:** Best model pipeline.

Description	Results
Pipeline ID	1394
Dataset ID	25
Number of selected features	31
Model	Extra Trees
Hyperparameters	*max_depth*=18, *n_estimators*=140
RMSE	2240.0833 (1)
%RMSE	16.8847 (1)
MAE	1276.9389 (1)
%MAE	9.6249 (1)
R2	0.8263 (1)
COD	16.0903 (1)
Average ranking	1

### Model interpretability analysis

#### Model based global feature importance.

**Fig 10:**
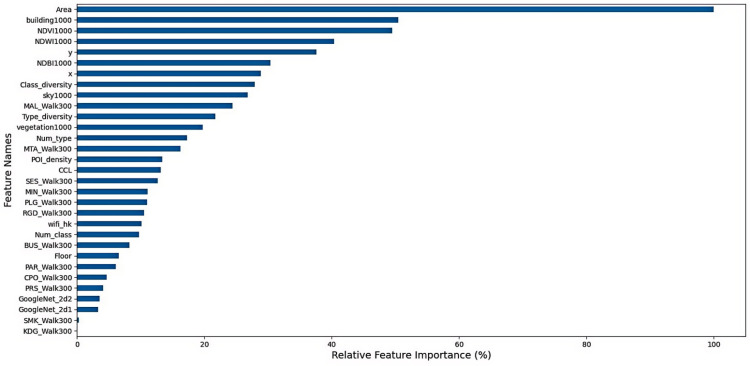
Relative feature importance.


Fig 10 shows the relative feature importance of selected features. The feature importance is calculated based on the Extra Tree model, and then the relative importance of feature i is calculated with the equation:

$$\begin{array}{c} \begin{array}{c} RI_{i}=\frac{f_{i}-f_{min}}{f_{max}-f_{min}}\times 100\% \end{array} \end{array}$$ (17)

where $f_{i}$ is the Extra Tree-based feature importance of feature i. $f_{max}$ and $f_{min}$ are the maximum and minimum feature importance, respectively. The floor area is the most significant feature, and the reason could be that floor area is closely related to the living comfort of residences, especially in densely populated cities like Hong Kong. Northing (*y*) and easting (*x*) are ranked 6^th^ and 9^th^, respectively. The geographic location of properties matters in determining the prediction accuracy, as suggested by [92]. POI class and type diversity are ranked 7^th^ and 11^th^, indicating that diversified distributions of POI places around the property could affect the housing price. Walking accessibility to MAL, MTA, and SES are the top 3 important features among all POI accessibility variables. Half of the top 10 significant features are image features, including building view (53.4%), NDVI (45.3%), NDBI (44.8%), NDWI (41.0%), and sky view (26.9%). The results show that images play significant roles in predicting housing prices.

We use the best pipeline for model performance comparison to further distinguish between the roles of different image features. The baseline experiment excludes all non-image features. Three experiments are conducted by feeding only one image type into the baseline experiment step by step. Finally, we train the pipeline with all image features. Due to the randomness of the Extra Tree model, the number of experiments for each type is set as 100. The average performances and rankings of each experiment type are provided in Table 9. According to the average ranks, the remote sensing (RS) image contributes the most to prediction accuracy among the three image types. The model incorporating all image features has the highest average ranks. It proves that the three types of images can characterize housing prices from different perspectives, and it is necessary to consider incorporating images to improve housing price prediction accuracy.

**Table 9:** Model performance in different combinations of image sources.

Experiment	RMSE	%RMSE	MAE	%MAE	R2	COD	Average ranks
Baseline (B)	2349.43 (5)	17.71 (5)	1324.14 (5)	9.98 (4.5)	0.809 (5)	16.55 (4)	4.8
B+SVI	2323.10 (4)	17.51 (4)	1303.76 (3)	9.83 (3)	0.813 (4)	16.38 (3)	3.5
B+RS	2313.15 (3)	17.44 (3)	1291.91 (1)	9.74 (1)	0.815 (3)	16.20 (1)	2.0
B+HP	2303.81 (2)	17.36 (2)	1323.58 (4)	9.98 (4.5)	0.816 (2)	16.57 (5)	3.3
B+SVI+RS+HP	2273.42 (1)	17.14 (1)	1299.90 (2)	9.80 (2)	0.821 (1)	16.38 (2)	1.5

#### SHAP based local feature importance.

**Fig 11:**
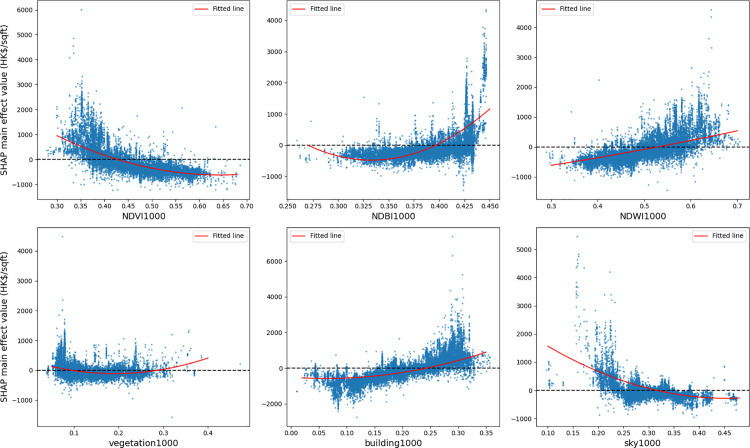
Nonlinear associations between image features and housing prices.

The relationships between image features and housing prices are depicted in Fig 11. Each blue point represents a sample of a housing unit, and the red lines are the fitted curve using the polynomial fitting method. The visual features of house photos do not have actual meanings and, therefore, are excluded from the analysis. The remote sensing and view index exhibit unique nonlinear associations with housing prices. The NDVI has a nonlinear and negative effect on housing prices, falling by 3000 HK$/sqft within the range of 0.30~0.67. The NDBI shows a nonlinear and positive impact on the housing price, which increases rapidly when the NDBI is larger than 0.425. The housing price increases linearly by about 2000 HK$/sqft when NDWI increases from 0.30 to 0.70. For VVI, the housing price is unchanged within the range of 0.1~0.3 and then increases when the index exceeds 0.3. BVI has a similar trend with NDBI, and the price increases by about 4000 HK$/sqft when BVI is within the range of 0.05~0.35. SVI has a negative and nonlinear effect on housing prices. The price decreases when SVI increases from 0.15 to 0.25, followed by a slowly decreasing trend when SVI is larger than 0.25.

**Fig 12:**
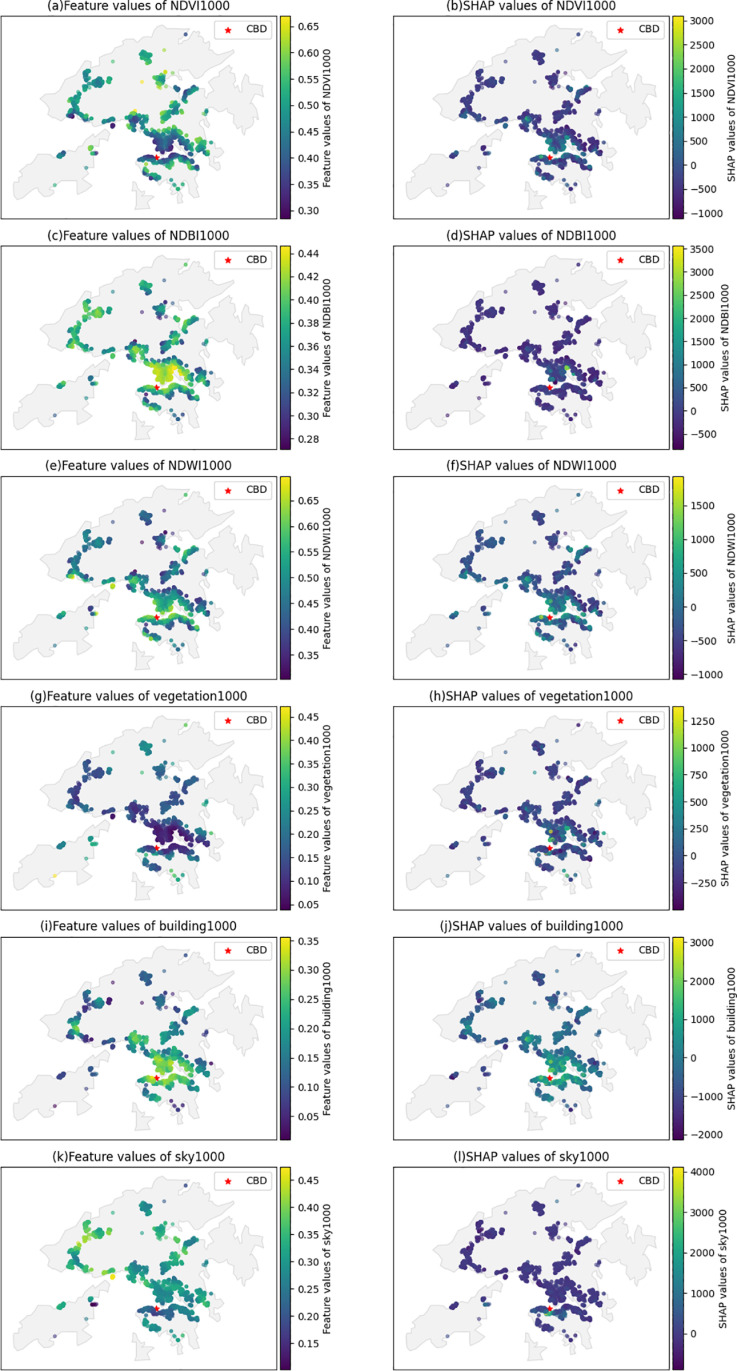
Spatial distribution of feature value and SHAP values of image features.

To further explain the effects of remote sensing and view index on housing prices, the spatial distribution of feature values and corresponding SHAP main effects are illustrated in Fig 12. The location of CBD is identified as Central and marked as a red star. Housing units near CBD are characterized by lower NDVI and VVI, higher NDBI and BVI, higher NDWI, and lower SVI. The urban greenness distribution is unequal in Hong Kong (Figs 12a and 12g): the central city has less greenness than other areas. The effects of NDVI and VVI vary significantly from the central city to the suburbs (Figs 12b and 12h). Higher NDBI and building view index contribute to higher housing prices (Figs 12c, 12d and Figs 12i, 12j), possibly due to areas with high building density having more amenity facilities and job opportunities. As Hong Kong is a coastal city, housing units along the coastal lines have higher NDWI and enjoy more sea view premium on the housing prices (Figs 12e and 12f). The sky view of housing units near the CBD is lower than in other areas (Fig 12k); however, it positively contributes to housing prices (Fig.12l).

### Practical implications of this study

Given the model interpretability analysis results, the implications for stakeholders involved in the real estate industry are provided: (1) **public authorities** can use the proposed housing price prediction model to formulate a formal property valuation model by considering multi-source image data fusion. The valuation model could be used in multiple government-led application scenarios: property tax estimation, dispute resolution about urban renewal compensation determination, and housing affordability assessment, etc. (2) **urban planners** can have an in-depth understanding of how urban infrastructure, as well as urban green and blue space, affects housing prices. The findings will enable the planners to make necessary adjustments to facilitate balanced urban development. For instance, POI diversity and accessibility are more important than POI density in determining housing prices in Hong Kong. Urban planners could be committed to increasing the diversity and accessibility of amenity facilities to meet the community’s evolving needs. The unevenly distributed urban greenness has been identified in Hong Kong, and urban planners should prioritize vegetation development in the urban centre to ensure green justice. (3) **real estate developers** should pay more attention to site planning and architectural design (e.g., floor area ratio optimization) to improve residents’ living conditions since the floor area is the most significant feature. Understanding the positive and negative effects of housing price determinants is necessary for real estate developers to help them evaluate potential development sites, conduct feasibility studies for new projects, and make data-driven decisions about investment opportunities. For instance, the project sites with lower NDBI and higher NDVI could be considered unpromising and lack investment potential in Hong Kong.

### Limitations and future research directions

This study has limitations: (1) The feature configuration parameter types and candidates used in this study are limited. Future studies can use more feature configuration parameters, such as diversified accessibility measures and CNN types for image extraction. Multiple hyperparameter optimization methods could be used to find the best feature combination instead of the time-consuming grid search used in this study; (2) There is a large computational demand for image feature processing and pipeline execution, which could consume much time for large-scale image data and generated pipelines. The potential bottlenecks for improving the computational efficiency of the proposed approach include CPU/GPU capability and training speed. Therefore, the approach could be further improved by enhancing the scalability in terms of hardware (e.g., better and more high-performance CPU/GPUs) and software (e.g., more efficient computing strategies); (3) The effectiveness of multi-source image data may vary significantly across different regions, and it would make the findings more convincing by conducting more experiments across diverse areas to analyze the generalizability of the findings and adaptability of the proposed framework. Future studies could use datasets in multiple regions to examine and compare the effects of multi-source images on housing prices.

## Conclusions

Using fine-scale housing transaction data in Hong Kong, this paper proposes an enhanced ML framework for residential property valuation with multi-source image fusion, including exterior estate photos, street view images, and remote sensing images. The research results show that different feature configuration parameters can significantly affect model performances. Formulating a series of datasets and searching for the optimal feature configuration parameters is necessary to improve the overall model performance. The MRMR-based feature selection method can effectively determine the optimal feature set. Extra Tree performs significantly better than others.

Model interpretability analysis is conducted based on the optimal machine learning pipeline, and the results prove that image features play significant roles in determining the prices. Half of the top 10 significant features are image features, including building view (53.4%), NDVI (45.3%), NDBI (44.8%), NDWI (41.0%), and sky view (26.9%). Incorporating image features into prediction models can improve the accuracy of housing price prediction, increasing the R squared from 0.809 to 0.821. Nonlinear and positive associations exist between housing prices and NDBI, NDWI, vegetation and building view. NDVI and sky view have nonlinear and negative associations with housing prices. The spatial distribution patterns of image feature values and corresponding SHAP main effects vary significantly from the city centre to the suburbs. Housing units near the centre are characterized by (1) lower NDVI, sky view and vegetation view index and (2) higher NDBI, NDWI, and building view index. This study provides practical implications for real estate stakeholders, including public authorities, urban planners, and developers. Future research directions could emphasize diversified feature configuration parameters, efficient computing strategies, and more case studies for validation.
